# Temperature Anomalies and Mortality Events in Marine Communities: Insights on Factors behind Differential Mortality Impacts in the NW Mediterranean

**DOI:** 10.1371/journal.pone.0023814

**Published:** 2011-09-12

**Authors:** Carolina Crisci, Nathaniel Bensoussan, Jean-Claude Romano, Joaquim Garrabou

**Affiliations:** 1 UMR 6540 - DIMAR CNRS - Université de la Méditerranée, Centre d'Océanologie de Marseille, Station Marine d'Endoume, Marseille, France; 2 IPSO FACTO, SARL, Pôle Recherche Océanologie et Limnologie, Marseille, France; 3 UMR 6134 CNRS - Université de Corse, Laboratoire Systèmes Physiques pour l'Environnement, Ajaccio, France; 4 Institut de Ciències de la Mar (ICM-CSIC), Barcelona, Catalonia, Spain; National Institute of Water & Atmospheric Research, New Zealand

## Abstract

Two large-scale mass mortality events (MMEs) of unprecedented extent and severity affecting rocky benthic communities occurred during the summers of 1999 and 2003 along the coasts of the NW Mediterranean Sea. These mortality outbreaks were associated with positive thermal anomalies. In this study, we performed an analysis of inter-regional and inter-annual differences in temperature (T) conditions associated with MMEs of the red gorgonian *Paramuricea clavata* by analyzing high resolution T time series (hourly records for 3 to 8 years) from four regions of the NW Mediterranean with differing hydrological conditions and biological impacts. High resolution records allowed a detailed analysis using classical and new descriptors to characterize T anomalies. We were able to determine that the MMEs were triggered by two main types of positive thermal anomalies, with the first type being characterized by short periods (2 to 5 days) with high Mean T reaching more than 27°C in some regions and being associated with high intra-day and intra-period variability, while the second type of anomaly presented long duration (near one month) at warm T (24°C) with low intra-period variability. Inter-regional patterns arose; some regions displayed both types of anomalies, while others exhibited only one type. The results showed that T conditions should be considered as the main factor that explains the observed inter-regional and inter-annual differences in mortality impacts. In explaining these differences, the late timing of T anomalies, in addition to their magnitude was found to be determinant. Finally, by combining thermotolerance experimental data with the maximal T stress conditions observed in the four regions, we were able to determine the differential risk of mass mortality across regions. We conclude that expanding high resolution T series is important for the development of sound management and conservation plans to protect Mediterranean marine biodiversity in the face of climate change.

## Introduction

Coastal marine ecosystems harbor high biological diversity and are among the most productive systems in the world [1], [2]. These ecosystems are subjected to high levels of anthropogenic pressure, which could have serious implications for the well-being of societies dependent on these ecosystems for goods and services [3].

Overexploitation has been recognized as the major threat to marine ecosystems causing the decline of a number of target species and changes in the structure of food webs [4], [5]. However, pollution, invasive species, alteration and loss of habitats and, more recently, global climate change have also been reported to have significant effects in marine ecosystems [6], [7], [8], [9], [10].

The analysis of climate change impacts presents a unique challenge for conservation biology because they affect large spatial scales and because they are not easily alleviated by local management actions [11]. Likewise, these impacts affect most levels of biological organization: from population and life-history changes to shifts in the species composition and in the structure and function of ecosystems [10], [12]. Therefore, research efforts focused on providing meaningful data for the development of management plans are urgently needed to enhance the resilience of ecosystems facing current environmental changes [8], [13].

In the NW Mediterranean (NWM) Sea, recent studies have demonstrated a clear warming trend during the last century and the enhancement of stratification conditions during summer periods in the last 30 years [14], [15], [16], [17]. In this region, warming has been found to be associated with shifts in species distributions [18], [19] and mortality events observed during the last 30 years [20], [21]. In particular, two recent large-scale (>1000 km of coastline) mass mortality events (MME) of approximately 30 macro-benthic species including sponges, cnidarians, bivalves, ascidians and bryozoans, occurred during the summers of 1999 and 2003 along the coasts of Spain, France and Italy. In 2006 and 2008, mortality events of a minor extent and severity were also documented in the NWM region [22], [23], [24]. All these events were associated with positive thermal anomalies [20], [21], [24], [25].

An analysis of the biological impacts of mentioned MMEs has revealed differential responses among species and their populations at all spatial scales considered [21]. At the local level, colonies can show contrasting responses, ranging from severe to a complete absence of injuries. Within regions, populations can display low to high mortality, and there is a clear decrease of impact with depth [26]. As an example, different red coral (*Corallium rubrum*) populations from the same region presented from 5% to 80% of affected colonies [27]. This magnitude of differences has also been observed at the inter-regional level [21]. Finally, the same regions affected by MMEs in different years exhibited differential impacts, both in magnitude and the depth range affected [20], [21], [25], [26].

In this study, we present an analysis of inter-regional and annual differences in T conditions associated with MMEs by analyzing high resolution T time series from four regions of the NW Mediterranean Sea with differing hydrological conditions [22] and biological responses [21]. The characterization of the thermal conditions of different regions and years that displayed mass mortality events and the analysis of corresponding impacts in the red gorgonian *Paramuricea clavata* populations, allowed for the first time the study of the relation of regional temperature conditions with observed impacts at the population level. High resolution T records allowed a detailed analysis using classical and new descriptors. Moreover, the results allowed the discussion of the potential role of temperature conditions and biological factors (e.g. acclimatization, local adaptation) that may underlie the differential impacts of the MMEs.

## Materials and Methods

### 2.1 Study area

**Figure 1:**
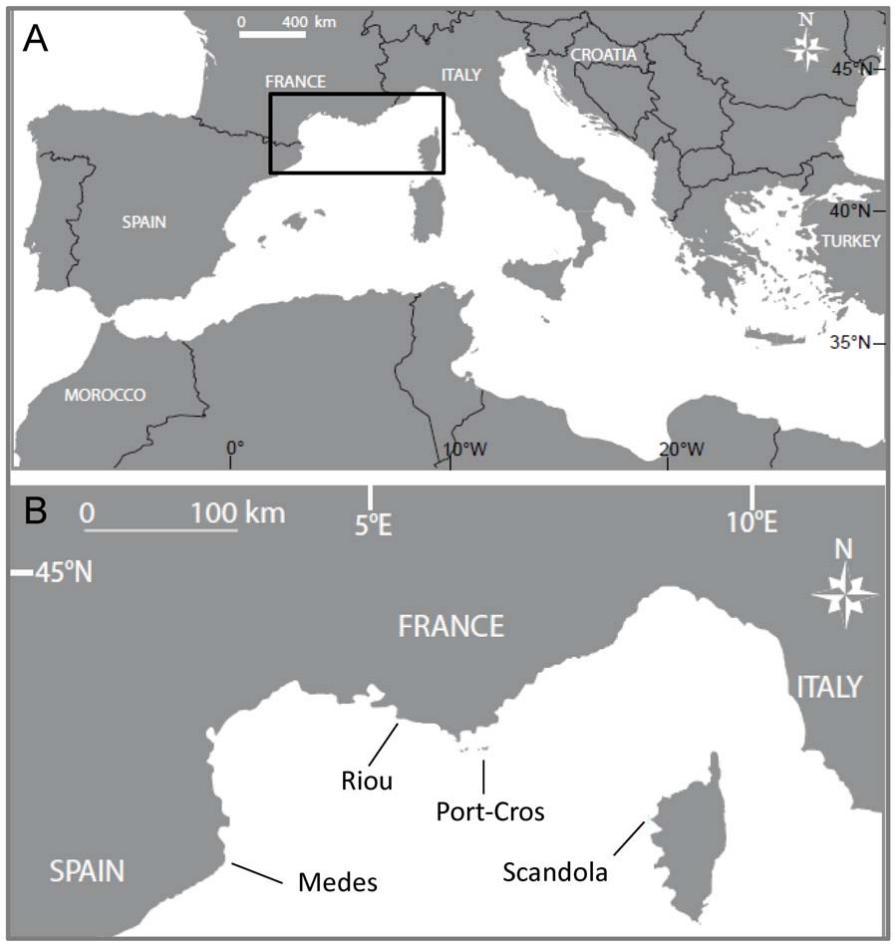
The study area. Northwestern Mediterranean Sea showing the limits of the study area (a) and detail of the NW Mediterranean with the four study regions (b).

The study was conducted in four locations of the NW Mediterranean basin (Fig. 1a), which were the following, from west to east: Parc Natural del Montgrí, Illes Medes i Baix Ter (L'Estartit, Spain); Riou (Marseille, France); Parc National de Port-Cros (France); and Reserve Naturelle de Scandola (Corsica, France) (Fig. 1b). These regions shared the common feature of NW Mediterranean waters of being characterized by a marked seasonality. From late autumn to winter (December–March), the seawater T slowly declines, reaching a minimum in March of approximately 13°C before increasing slightly until the formation of the thermocline [28]. Although they present similarity in their annual T cycles, during summer, the four regions present very distinct hydrographic conditions [22]. Riou exhibits the coldest conditions from depths of 5 to 40 m, while Scandola is the warmest site in its subsurface waters, and Medes and Port-Cros show intermediate conditions. From depths of 15 to 35 m, the warmest T occurs at Medes and Scandola, with Port-Cros being in an intermediate position [22]. With respect to the variability of the summer thermal regime, inter-regional differences are also observed. Riou is the most variable site from depths of 5 to 40 m because of the occurrence of upwelling, while Medes exhibits the highest variability at 40 m because of the recurrent downwelling. Finally, Port-Cros and Scandola display the maximum variability at 25–30 m because of oscillations of the thermocline that settles around these depths [22].

### 2.2 Temperature measurement strategy

In each region, T records were registered by *in situ* Stowaway Tidbits autonomous sensors set up in sites exposed to dominant winds and currents.

The recorded period was from 1999 to 2006. Records began in June 1999 at Riou and Port-Cros, in July 2002 at Medes and in April 2004 at Scandola. Since 2004, measurements have been standardized at all regions to collect hourly records. Previously, T measurements had been recorded every 2 hours, and these time series were interpolated using an exact interpolation technique to obtain a set of synchronous hourly data. T data were not available for all years within each region; the available periods of T measurements at each depth are shown in Table 1. These temperature series were previously used to characterize temperature regimes in the four studied regions ([22]).

**Table 1:** Availability of temperature data.

Geographic region	10 m	25 m
Parc Natural del Montgrí, Illes Medes i Baix Ter (L'Estartit, Spain)	2003–2006*	2003–2006
Riou (Marseilles, France)	1999–2006	1999–2006
Parc National de Port-Cros (France)	1999, 2001–2006	1999–2000, 2002–2006
Reserve Naturelle de Scandola (Corsica, France)	2004–2006	2004–2006

Available temperature data for the four study regions in the northwestern Mediterranean Sea at 10 and 25 m depths (*: temperature at 15 m depths was analyzed instead of temperature at 10 m). The selected depths include the depth range in which the most severe impacts of MMEs were observed.

### 2.3 Biological data collection

Biological surveys were conducted in the four study regions to investigate relationships between temperature conditions and population responses. For this purpose, we chose populations of the red gorgonian *Paramuricea clavata* as model species because the largest dataset was available for this species, and it was one of the most affected during the MMEs [20], [21], [25]. Furthermore *P. clavata* is considered one of the key species of Mediterranean coralligenous assemblages ([29],[30]). Since other macrobenthic species affected by the MMEs showed similar inter-regional and inter-annual pattern of mortality to *P. clavata*, the patterns obtained for this species may be considered representative of the MMEs impacts for other species as well ([20],[21], authors unpublished data). For all these reasons we contend that the use of *P. clavata* provide an excellent model for the analysis of the relationships between temperature conditions during temperature anomalies and biological impacts.

During the surveys, the percentage of recent tissue necrosis (i.e., denuded axis or recent epibiosis) was quantified in at least 100 colonies present at each site and depth surveyed.

We considered a colony to be affected by mortality when it showed recent tissue necrosis over 10% of its surface. Finally, for each survey, the percentage of affected colonies was calculated as an indicator of the mortality impact (see [21] for further information). Surveys were conducted on an annual basis at 2–7 sites within each study area. In cases where a MME was observed, the number of sites was increased when possible to better quantify the mass mortality impacts. More than 20.000 colonies were analyzed within the four study regions. We calculated the percentage of affected colonies within each region at every year that presented mass mortality events and inside each region we averaged the values of the years that not displayed mortality outbreaks. Percentages were calculated separately for 10 and 25 m depth ranges. In Port-Cros and Scandola, the surveys only concerned the 25 m depth because at 10 m, *P. clavata* populations are absent or show low abundance [26], [31].

Kruskal-Wallis analysis was used to test for differences of mortality rates between all regions, years and depths. Multiple comparisons were studied through Mann-Whitney tests to determine specific differences between pairs of data. Nonparametric tests were selected because of the absence of normality and homoscedasticity in the dependent variable. The Kruskal-Wallis and Mann-Whitney tests were computed using PAST software (version 1.82b, [32]).

### 2.4 Characterization of temperature anomalies related to mass mortality events (MME)

To characterize the temperature conditions of years associated with mortality events in each region, we combined classical and new descriptors to retain information on the magnitude, variability and duration of T anomalies, as well as the timing of the anomalies during the summer period (see below).

In the analysis, the period between 1^st^ July and 30 September was arbitrarily considered as the summer period. Likewise, the depths considered were 10 and 25 m (12 and 24 m at Riou and Port-Cros in the 1999–2003 period, hereafter referred to as 10 and 25 m, respectively), which correspond to the suprathermoclinal and intermediate thermoclinal levels, respectively [22]. We selected this period and these depths because the MMEs displayed the most severe impacts under these conditions [20], [21], [23], [25].

Finally, we distinguished two types of years: those associated with mortality events in at least some of the studied regions (hereafter YMMEs), which included 1999, 2003 and 2006; and those presenting no mortality event signals (hereafter YNMMEs), which included 2000, 2001, 2002, 2004 and 2005. For all of the analyzed statistics, for each location and depth, each YMME was analyzed separately, while YNMMEs were studied together, averaging statistical values. Representation and analysis of the data were performed using SigmaPlot (version 10.0) and PAST (version 1.82b, [32]) software, respectively.

#### 2.4.1 Mean T, maximum T and coefficient of variation of the summer period

The Mean T, maximum T (Max T) and coefficient of variation (CV) were calculated to search for differences between YMMEs and YNMMEs and within YMMEs. The CV (summer standard deviation×100/summer Mean T) was chosen as the measure of variability because it is the percentage of the degree of variability and can be interpreted independently from the mean.

#### 2.4.2 Mean and CV of time intervals with the highest Mean T

Consecutive episodes of 2, 5, 10, 15, 30 and 40 days (taking 24 consecutive hours as a day, 48 consecutive hours for two days, and so on) with the highest Mean T were retained. For each year, region and depth, there was a unique corresponding consecutive period of a specific length with the highest Mean T. Thus, the value of this Mean T and the corresponding value of the CV of each length period were retained with the aim of capturing the T magnitude and associated variability of the hottest periods of short, intermediate and long duration.

#### 2.4.3 Timing of periods with the highest Mean T

The timing of the above-mentioned 15- and 40-day periods was analyzed. Timing refers to the point in time in the summer when these periods occurred. For the 15-day episodes, summer was divided into 6 two-week summer periods: 1^st^–15 July, 16–31 July, 1^st^–15 August, 16–31 August, 1^st^–15 September and 16–30 September. Then, the 15-day episodes with highest Mean T were associated with the two-week period in which most of the episode occurred. For the 40-day episodes, the summer period was divided into months: July, August and September. Again, the 40-day episodes with highest Mean T were associated with the month in which most of the episode occurred. By analyzing the timing of the temperature anomalies, we intended to explore the response of affected species to similar temperature stresses occurring at different times during the summer.

### 2.5 Ordination of YMMEs and YNMMEs through T statistics

The analyzed statistics (except those on temperature anomalies timing) were ordered through Principal Component Analysis (PCA) with the aim of synthesizing the information provided by the different T indicators. Two analyses were performed, one for the 10 m depth and another for the 25 m depth. In addition to the analyzed statistics described above, three further variables were considered to perform the PCA. We included, in one side, the total duration (as the proportion of summer time) inside the [24]–[25] °C T class and the longest consecutive duration inside this class. These statistics were considered since it was documented that during 1999 summer, long duration near 24°C occurred at Riou and at Port-Cros ([22], [31]). In the other side we included the average CV of the 5 hottest summer days. It was calculated averaging the CV value corresponding to the 5 summer days with the highest Mean T. This indicator was included with the aim of considering variability of the thermal regime at shorter time scales (e.g. inside days). Overall, a total of 15 variables were available to perform the analyses, but because of the very high correlation between some of them (Pearson correlation coefficient >0.8), redundant variables were removed to perform PCAs. Finally, a total of six and eight variables were retained to perform the 10 and 25 m depth PCA, respectively. For the 10 m depth PCA the retained variables were the Mean T of the 5 and the 40 consecutive days with highest mean T (Mean_T_5 and Mean_T_40 respectively), the CV of the 5 and the 40 consecutive days with highest mean T (CV_5 and CV_40 respectively), the average CV of the 5 hottest summer days (Mean_CV_5) and the total duration inside the [24]–[25] °C T class (Dur_24). For the 25 m depth PCA all the variables retained for the 10 m depth PCA, the CV of the 15 consecutive days with highest mean T (CV_15) and the longest consecutive duration inside de [24]–[25] °C T class (Max_cons_dur_24) were analyzed.

As for the previously calculated statistics, YNMMEs were considered together, while YMMEs were studied separately.

### 2.6 Confronting thermotolerance experiment results and field T conditions

The available information from the experimental results on the thermotolerance of NWM rocky benthic species (Table 2) was contrasted with the most severe field T conditions observed in the four studied regions. An inverse second-order regression (f = y0+(a/x)+(b/x^2^) was fitted to the Mean T of increasing time intervals (from 2 to 40 days) with the highest Mean T with the aim of obtaining a domain of possible conditions in each region given the available datasets and comparing them with the upper thermotolerance limits from experimental data. Given that points located in domains below the regression curves indicate species at risk under actual conditions, we suggest that differences with respect to the distribution of experimental data above and below the regression curve could provide clues related to the differential risk of mortality among regions.

**Table 2:** Availability of experimental data.

Species	Tested T (°C)	References
*Cladocora caespitosa*	24, 26,	[56]
*Corallium rubrum*	24, 25, 27	[38], [39]
*Eunicella singularis*	24	[57]
*Oculina patagonica*	24, 26,	[56]
*Paramuricea clavata*	23, 24, 25, 25, 27	[16], [37], [39], Crisci et al. unpublished data

Data from experimental work on T effects on mortality (necrosis) of the NWM rocky benthic species used to produce Figure 7.

## Results

### 3.1 Biological data

**Figure 2:**
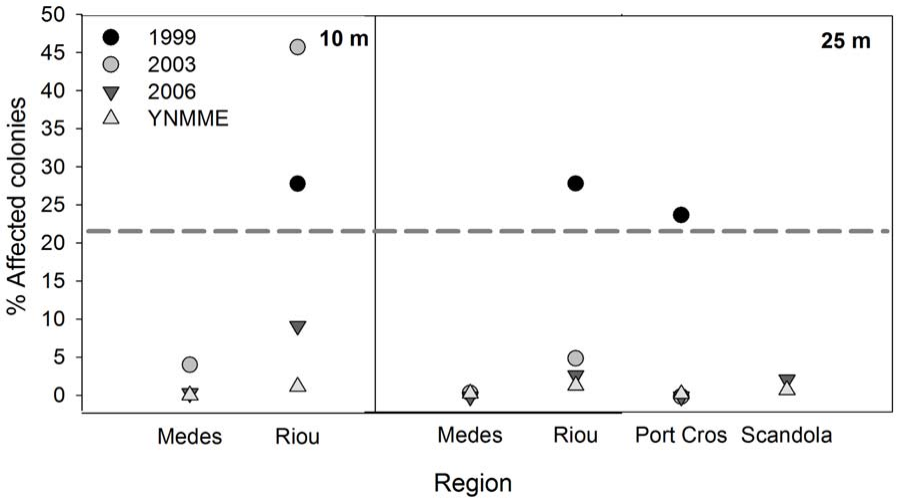
Mortality of *Paramuricea clavata*. Percentage of affected colonies with ≥10% of the colony surface showing recent necrosis (denuded axis or recent epibiosis) for *Paramuricea clavata* populations in the four study regions of the northwestern Mediterranean sea at 10 and 25 m depths during 1999, 2003, 2006 and YNMMEs. The cases with the highest rates of necrosis are those above the grey dashed line. Within this group of data there were no statistical differences in mortality rates (p-value>0.05), while % of affected colonies showed statistical differences with the remaining years, regions and depths (p-value<0.5). There was an exception for Port-Cros in 1999 at the 25 m depth, which did not present differences with Riou in 2006 at the 10 m depth.

Clear differences were found in the mortality rates associated with different years, regions and depths, and these differences were significant (Kruskal-Wallis p-value<0.05). The highest mortality rates were found at Riou in 1999 at 10 and 25 m depths, at Riou in 2003 at the 10 m depth and at Port-Cros in 1999 at the 25 m depth, which all presented between 23 and 46% affected colonies (Fig. 2). Multi-comparison analyses did not indicate significant differences among these observations (p-value>0.05). These comparisons also showed that the cases with high mortality rates presented significant differences compared with all remaining cases, which experienced low to zero mortality rates (p-value<0.05). However, there was an exception for Riou in 2006 at the 10 m depth, which did not present differences with Port-Cros in 1999 at the 25 m depth. At this time and depth, Riou presented nearly 10% affected colonies, which was a value that was significantly higher than those of observations associated with low mortality rates (p-value<0.05, Fig. 2).

### 3.2 Characterization of the summer thermal regime of YMMEs

#### 3.2.1 Mean T, maximum T and coefficient of variation of the summer period

At 10 m depths, the highest summer Mean T was observed in 1999 at Riou and Port-Cros and in 2003 at Medes (Table 3). For this year at these regions, the highest Mean T values were more than 1°C warmer than those in all remaining years. In particular, this value for Riou in 1999 was almost 2.5°C warmer than for YNMMEs. Finally, Scandola did not present important differences between 2006 (the only YMME with T data available) and YNMMEs. The Max T was reached in 2003 at Medes, Riou and Port-Cros and in 2006 at Scandola. In this last region and year, the highest Max T of all regions and years was observed, reaching almost 28°C (Table 3). Additionally, either 2003 or 2006 was the year that presented the highest CV in all regions. The high values of the CV in Riou are remarkable in both YMMEs and YNMMEs in comparison with the other regions (Table 3).

**Table 3:** Classical T descriptors.

Region	Year	Mean summer T (°C)	Max. summer T (°C)	Summer CV (%)	Mean summer T (°C)	Max. summer T (°C)	Summer CV (%)
		10 m depth			25 m depth		
Medes	2003	22.3	25.5	8.4	18.3	24.5	15.2
	2006	21.6	25.0	10.1	19.6	24.9	14.3
	YNMME	21.2±0.7	23.4±1.1	4.6±1.4	19.5±0.3	22.9±0.9	9.9±3.3
Riou	1999	21.4	25.0	15.1	19.4	24.1	17.4
	2003	20.3	27.6	17.7	17.5	24.2	13.4
	2006	20.2	26.8	16.2	18.3	23.6	15.0
	YNMME	19.0±0.3	24.7±0.9	17.5±1.2	17.5±0.3	23.8±1.3	15.9±2.4
Port-Cros	1999	22.8	25.5	8.3	20.3	25.1	15.0
	2003	21.7	27.2	12.8	18.1	23.0	13.6
	2006	22.0	26.5	8.8	18.9	23.1	9.3
	YNMME	21.4±0.9	24.9±0.8	8.9±1.6	18.4±0.6	23.8±0.4	12.1±1.1
Scandola	2006	23.0	27.9	7.0	19.7	23.8	9.3
	YNMME	23.3±0.4	25.7±0.5	4.8±1.3	19.9±0.7	24.8±0.5	12.0±0.3

Mean T, maximum T (Max T) and coefficient of variation (CV) for the summer period of the four study regions for 1999, 2003, 2006 and YNMMEs (mean ± SD) at 10 and 25 m depths.

At the 25 m depth, as at the 10 m depth, the highest Mean T in Riou and Port-Cros were found in 1999 (1 to 2°C higher than other years). No remarkable differences were found among years for the other regions. However, in relation with the Max T, clear differences were found between Medes in 2003 and 2006 compared with YNMMEs (up to 2°C warmer) and Port-Cros in 1999 compared with all other years (1.3°C warmer). In contrast, in Scandola in 2006, the Max T was 1°C colder than in YNMMEs, indicating enhanced stratification, with warm T limited to shallow depths at this location. In Medes, 2003 and 2006 were more variable than YNMMEs, while 1999 and 2003 presented higher CVs than 2006 and YNMMEs in Port-Cros (Table 3).

#### 3.2.2 Mean T and CV of consecutive time intervals with the highest Mean T

**Figure 3:**
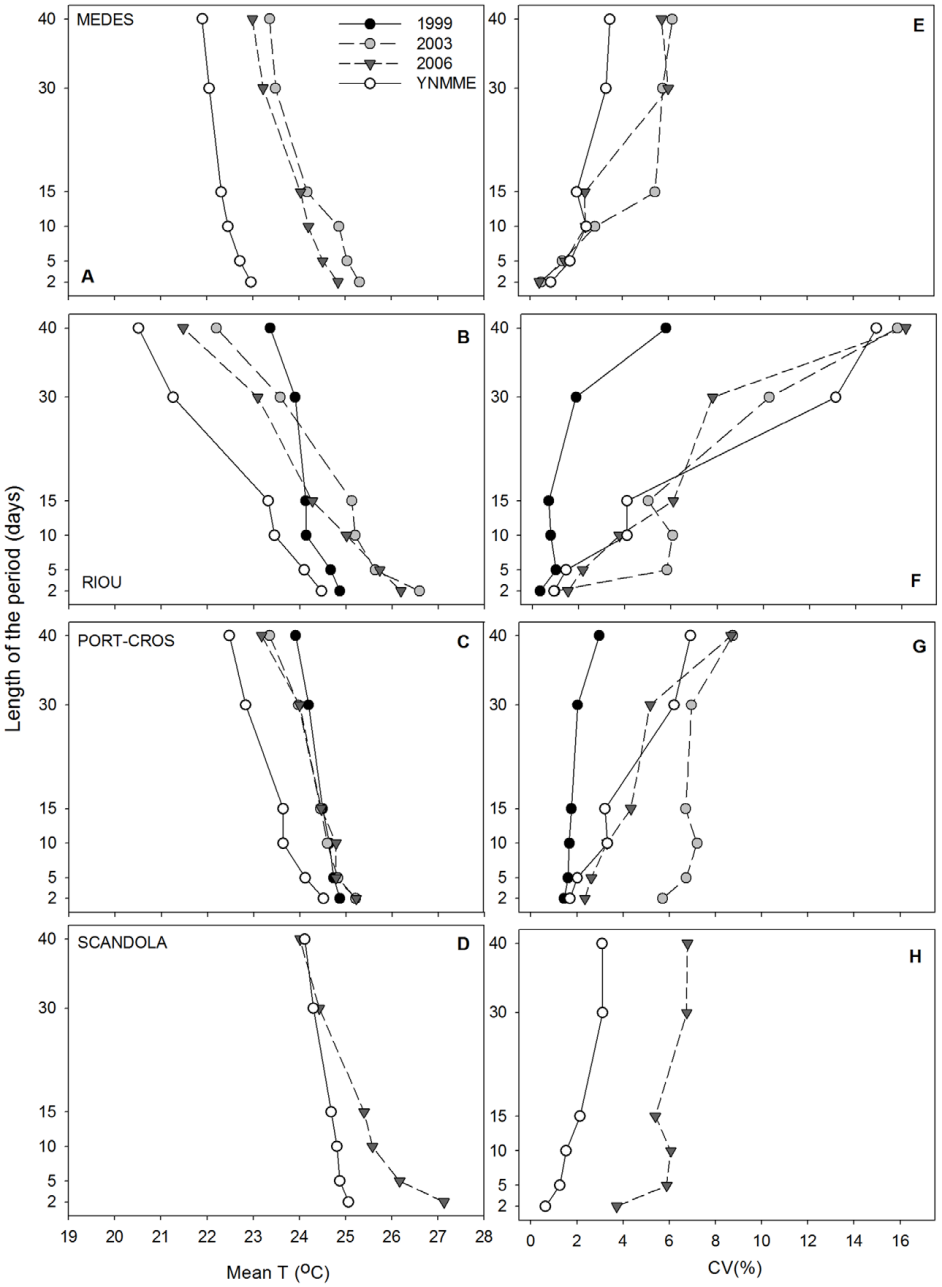
Mean T and CV of consecutive episodes with the highest mean T (10 m depth). Mean temperature (°C) and coefficient of variation (%) of consecutive episodes of 2, 5, 10, 15, 30 and 40 days with the highest mean temperature. Data are presented for the four study locations at 10 m depths.

In general, at the 10 m depth, YMMEs presented a higher Mean T than YNMMEs, regardless of the time interval considered (Figs. 3a–d). However, inter-annual and inter-regional differences were observed. Medes in 2003 and 2006 (Fig. 3a) and Riou and Port-Cros in 1999 (Figs. 3b and 3c) presented remarkable constancy throughout all time periods considered compared with YNMMEs, reaching a Mean T near 24°C in the longest periods and a T near 25°C in the shorter periods considered. On the other hand, clear differences with YNMMEs were also found in the Mean T for short periods at Medes and Riou in 2003 and 2006 and at Scandola in 2006, where the Mean T values recorded were approximately 2–3°C higher than the values found in YNMMEs (Figs. 3a, 3b and 3d).

Changes in the CV for the different time intervals were also observed during YMMEs at 10 m depth (Figs. 3e–h). In Riou and Port-Cros in 1999, the constancy in the Mean T over the different time intervals was reflected in a drastic decrease in the CVs compared with other years (10 and 5.5%, respectively, for the longest episodes) (Figs. 3f and 3g). For the other investigated years, Riou and Port-Cros showed CV values similar to those for YNMMEs, except in Port-Cros in 2003, where the CV for short periods was greater than for YNMMEs. In Medes and Scandola, YMMEs were characterized by an increase of the CV, especially when long periods of time were considered (Figs. 3e and 3h).

**Figure 4:**
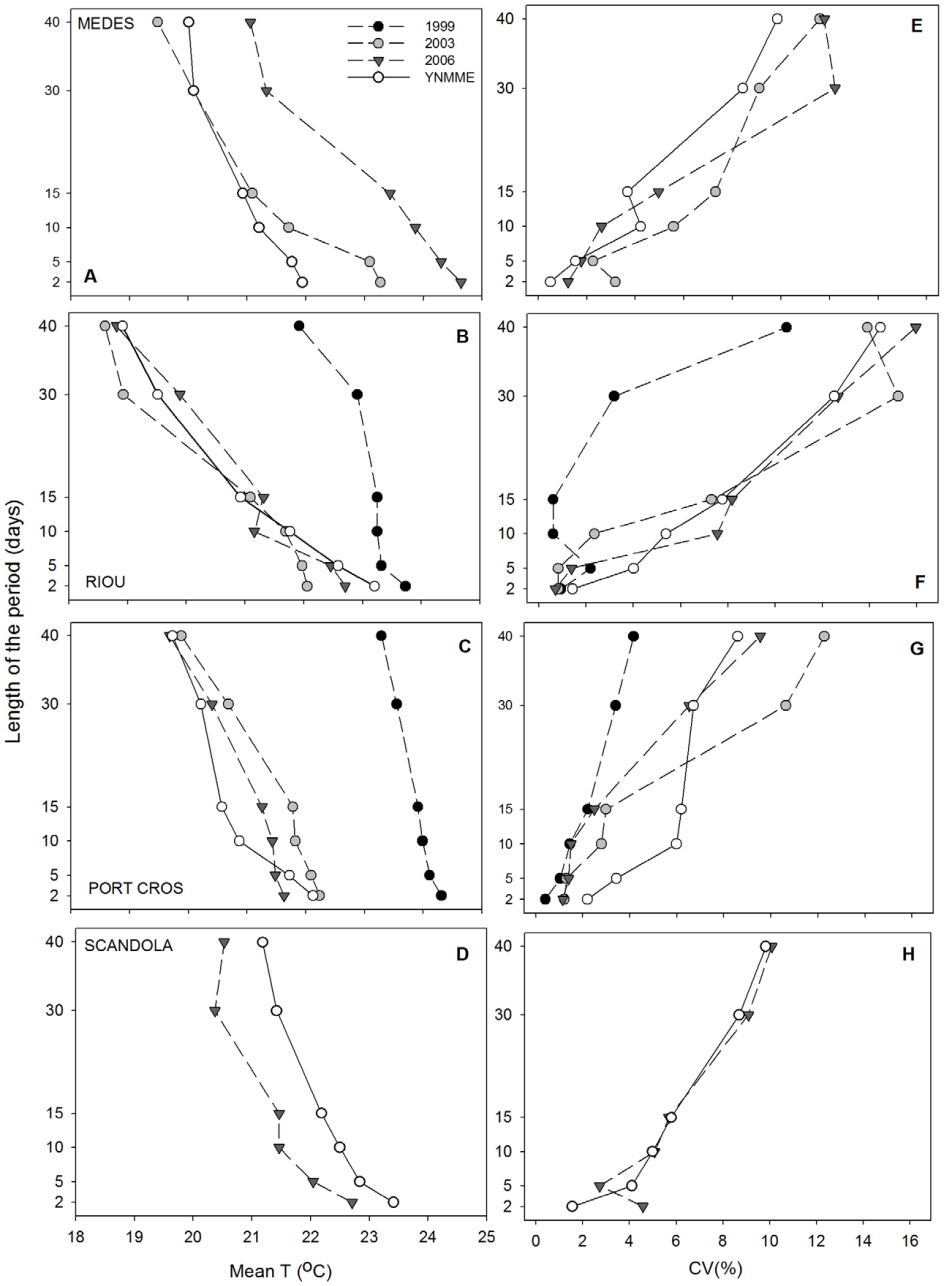
Mean T and CV of consecutive episodes with the highest mean T (25 m depth). Mean temperature (°C) and coefficient of variation (%) of consecutive episodes of 2, 5, 10, 15, 30 and 40 days with the highest mean temperature. Data are presented for the four study locations at 25 m depth.

At the 25 m depth, the Mean T of YMMEs versus YNMMEs did not differ as strongly, and it was warmer for all time periods examined only in Medes in 2006 and Riou and Port-Cros in 1999 (Figs. 4a–c). Medes in 2003 presented a higher T than YNMMEs, but only for short-length (≤5 days) episodes (Fig. 4a). All other years and regions did not present remarkable differences with YNMMEs (Figs. 4a–d). The most important differences compared with YNMMEs (2 to 4°C) were observed in Port-Cros in 1999, which presented a notable constancy in exhibiting a relatively high T throughout all time periods considered, reaching Mean T from 23.5°C to 24.5°C for the longest and the shortest periods, respectively (Fig. 4c). A similar pattern, though mainly concerning intermediate and long episodes, was observed in Riou in 1999 (Fig. 4b).

Because of the relative constancy in the 1999 Riou and Port-Cros thermal conditions, the CV was lower than that in all remaining years during intermediate and long episodes in Riou (Fig. 4f) and for long episodes in Port-Cros (Fig. 4g). The remaining years and regions did not differ significantly from YNMMEs (Figs. 4e–h).

#### 3.2.3 Timing of consecutive time intervals with the highest Mean T

**Figure 5:**
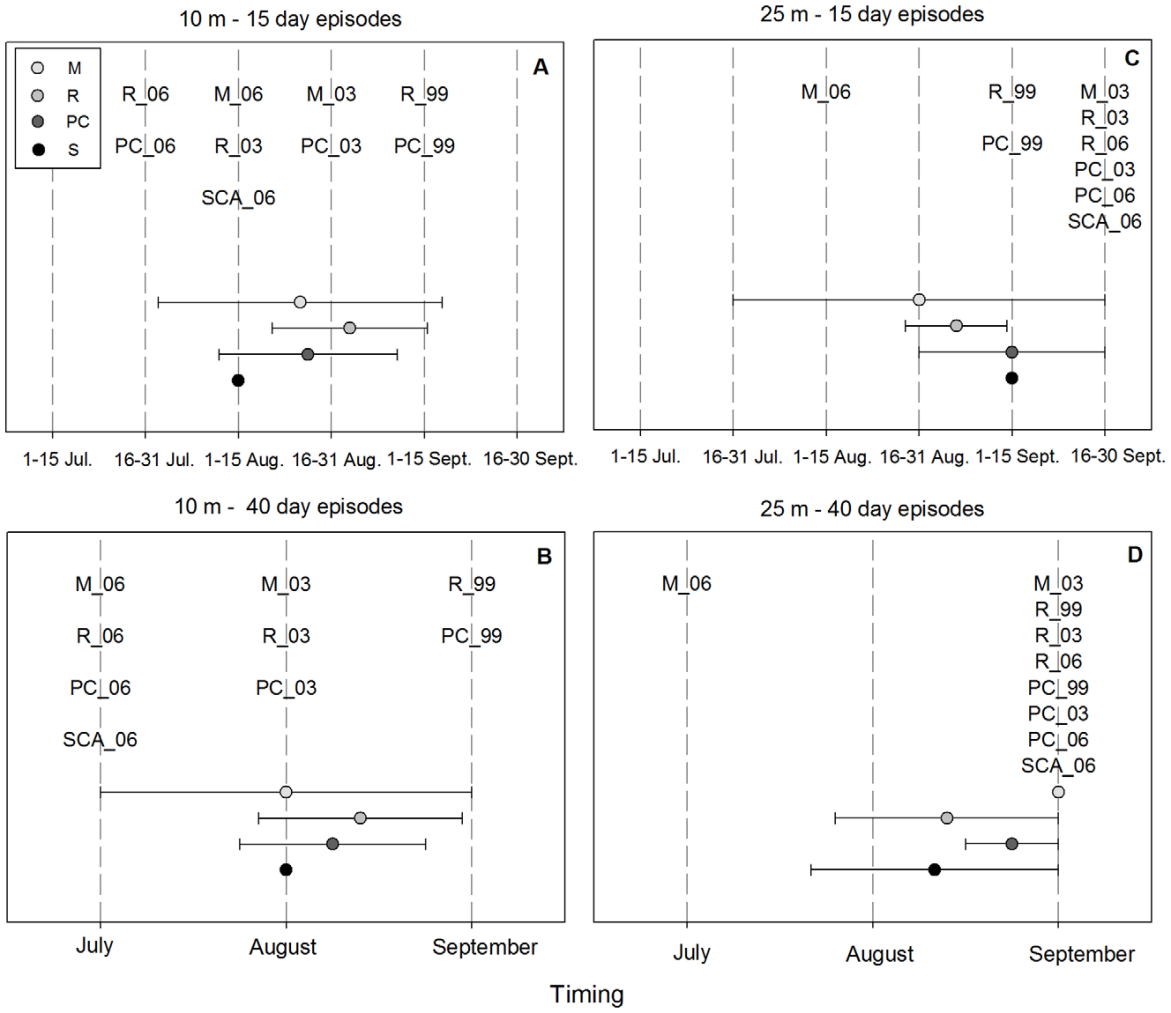
Timing of the 15 and 40 consecutive days with the highest mean T. Timing of consecutive episodes of 15 (a, c) and 40 days (b, d) with the highest mean T for 10 and 25 m depths. The average ± SD is presented for YNMMEs. Cases were ordered in the Y axis to facilitate data reading, but their position in this axis does not provide any information. M: Medes, R: Riou: PC: Port-Cros and S: Scandola.

At the 10 m depth, the 15-day episodes with the highest Mean T were relatively well segregated according to years (Fig. 5a). During 2006, these episodes occurred between the beginning and the middle of summer (16–31 July in Riou and Port-Cros and 1^st^ to 15 August in Medes and Scandola). In 2003, they occurred mainly in the middle of the summer period (1^st^ to 15 August in Riou and 16 to 31 August in Medes and Port-Cros), and in 1999, they occurred late in the summer period, between 1^st^ and 15 September (Fig. 5a). For YNMMEs, the warmest 15-day consecutive episodes occurred in August, though there was a great deal of variability depending on region (Fig. 5a).

When 40-day consecutive episodes were considered, 1999, 2003 and 2006 were again well segregated. These episodes occurred in July during 2006, in August during 2003 and in September during 1999. During YNMMEs, they were mainly distributed in the middle of the summer, although great variability was observed within some regions (Fig. 5b).

At 25 m, both 15- and 40-day episodes occurred first in Medes 2006 (between 1^st^ and 15 August and in July, respectively), while for all other YMMEs, episodes of both lengths occurred during September (Figs. 5c and 5d). YNMMEs presented intermediate and long hottest episodes, mostly at the end of the summer and always earlier than for YMMEs, although variability within some regions was observed (Figs. 5c and 5d).

### 3.3 Ordination of YMMEs and YNMMEs through T statistics

**Figure 6:**
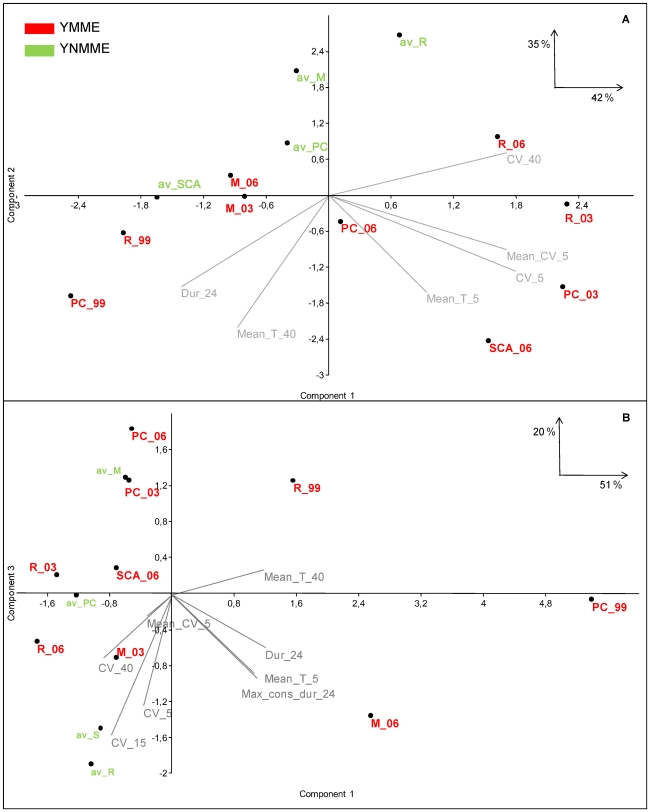
Principal Component Analysis representation. Representation of the first two axes of the Principal Component Analysis using T data from 10 (a) and 25 m depths (b). Two first axes were retained at both depths, accounting for 77 and 71% of data variability. The original T variables used to perform the analyses are represented in grey. Scandola in 2004 at the 10 m depth was not considered among the Scandola YNMMEs in the PCA because this location and year presented the particularity of reaching high temperatures (but not as high as for YMMEs at this location) with no mortality, so it affected the data ordination when considering all locations together. M: Medes, R: Riou: PC: Port-Cros and S: Scandola.

Considering the PCA for the 10 m depth, the first two axes accounted for 77% of the variance of the data. The first axis, which retained 42% of the variance, was useful for discriminating two main types of T anomalies. Riou and Port-Cros in 2003 and Scandola and Riou in 2006 were positively associated with this axis (Fig. 6a). Projection of the original T variables illustrates the summer thermal characteristics of these regions and years, which were associated with high T during short periods of time (five days) and with large hourly variability during the hottest summer days. High variability within short, intermediate and long hottest episodes was also a feature of these years. Negatively associations with the first axis were found for Riou and Port-Cros in 1999 and, with lower associated scores, Scandola in YNMMEs and Medes in 2003 and 2006. With the exception of Scandola in YNMMEs, these years represent a second type of thermal anomaly. This type of anomaly was characterized by long total and consecutive durations during warm T as well as by low variability at all time scales considered (Fig. 6a). The position of Scandola YNMMEs could be explained because the subsurface waters of this region are the hottest among the four regions (Table 3).

The second axis accounted for the characteristics of YNMMEs (with the exception of Scandola YMMEs), which were mainly low Mean T of short and large consecutive episodes with the highest Mean T (Fig. 6a).

At the 25 m depth, the first two axes accounted for 71% of the variance of the data (Fig. 6b). The first axis (51% of variability) distinguished the years that presented anomalous thermal regimes from those that did not. These years were 1999 for Port-Cros and Riou and 2006 for Medes and were characterized by a relatively long duration within warm T and relatively high T in short and long intervals with the highest Mean T. The second axis explained low data variability and did not clearly segregate YMMEs from YNMMEs. It was associated with variability related to different length episodes and separated regions inside YMMEs and YNMMEs (Fig. 6b).

### 3.4 Confronting thermotolerance experiment results and field T conditions

**Figure 7:**
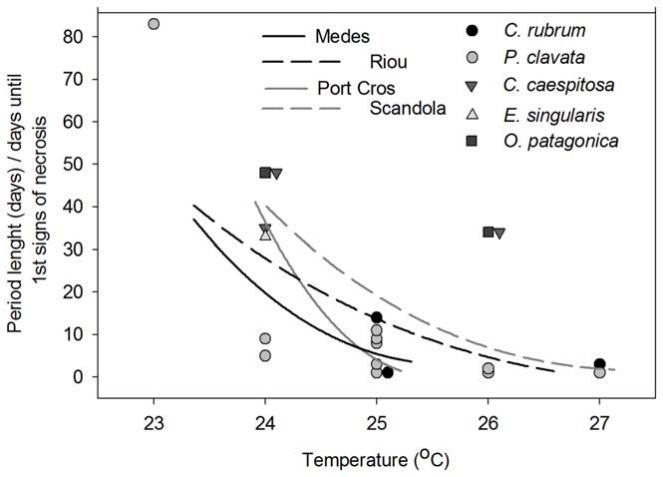
Field T versus experimental thermotolerance data. Inverse second order regression (f = y0+(a/x)+(b/x∧2) fitted to field T data (highest mean T of different length episodes) of the four study regions at 10 m depth (complete and dashed lines) and experimental results (day of 1^st^ signs of necrosis) from different rocky benthic species (grey and black symbols) obtained from the bibliography and the authors' unpublished data (see Table 2). For *E. singularis* and *O. patagonica* at 24°C and for *O. patagonica* at 26°C, the number of days until the 1^st^ signs of necrosis actually indicates the number of experimental days since no necrosis was observed until the last day. The aim of the figure is to represent a composite function over which, under experimental conditions, the species seems to tolerate field T regimes and under which species are affected by tissue necrosis.

The distribution of the experimental thermotolerance data points around the field T data curves provided clues related to the degree of vulnerability of the different species under recent T conditions in the 4 regions (Fig. 7). The experimental results indicated that short to moderate exposure (1 to 14 days) to 25°C and short exposure to 26 and 27°C (1 to 3 days) could lead to mortality (with the exception of the symbiotic species *C. caespitosa* and *O. patagonica*, which seem to be more resistant to these T conditions). These temperature conditions were attenuated in Medes and Port-Cros regarding exposure to 25°C, and there was very low or nonexistent exposure to 26 and 27°C at these sites. Conversely, these conditions were more frequent in Riou and Scandola for YMMEs, reaching longer durations at 25 and 26°C and, in the case of Scandola, also at 27°C (Fig. 7). Therefore, Medes and Port-Cros appear to represent the less risky of the investigated regions, while in contrast, Riou and Scandola appear to be the most risky regions in terms of higher chances of experiencing mortality.

## Discussion

In this study, we addressed for the first time the relationship of inter-regional and inter-annual differences in temperature conditions with the observed impacts in the macrobenthic populations of the NWM Sea during mass mortality outbreaks.

We were able to recognize two types of thermal anomalies that were likely to trigger an MME. The first type was characterized by short episodes (2–5 days of duration) with high Mean T, which were near 27°C in some regions, and high intra-day and intra-period (2–5 days) variability. The second type of anomaly presented long periods (30–40 days) with warm Mean T of approximately 24°C associated with low intra-period variability.

We found inter-regional and inter-annual differences in the occurrence and characteristics of the recorded anomalies. Riou and Port-Cros showed both types of anomalies: long term in 1999 and short term in 2003 and 2006. Furthermore, Medes displayed only long-term anomalies in both years in which anomalies were analyzed (2003 and 2006). Finally, in Scandola, a short-term anomaly was observed in the only year with anomalous T conditions (2006) for which data were available. It is worth noting that both in 2003 and in 2006, the two types of anomalies occurred simultaneously among regions, indicating that two of the largest heat-waves to ever peak over southern Europe [33], [34] resulted in differential anomalous warming conditions in the water column. These differences could be attributed to the distinct summer hydrological conditions found in the four study areas [22].

In Medes, there are recurrent downwellings (>40 m) during summer [22], which carries increased T further down in the water column, resulting in warm conditions over longer periods, but never reaching the elevated temperatures found in other study areas. In Riou and Port-Cros, the hydrological conditions can experience abrupt changes under the influence of Mistral (NWN) winds [35]. The lower frequency and shorter duration of these winds prevent the upwelling of deep, cold waters and the subsequent breakage of the thermocline, thus allowing the T to increase [22]. Scandola exhibits stable summer stratification conditions [22], and thus, the high air temperature and calm weather conditions recorded during the 2006 heat wave resulted in a significant T increase at the suprathermoclinal level. The occurrence of the long- and short-term anomalies recorded can likely be mainly attributed to the timing of calm conditions in the early-middle for 2003 and 2006, and late summer for 1999. The observed differences in the magnitude of both types of T anomalies were able to explain the differences in mortality rates observed in the field. Short periods of high T and long periods of warm T were associated with high mortality rates, being the attenuation of these characteristics (short periods of moderate T or shorter periods of warm T) linked to a significant decrease of mortality rates. Finally, years without thermal anomalies presented negligible mortality rates. Despite these relationships, it is important to precise that within the same region populations experiencing the same thermal regime presented different mortality impacts during MME ([21],[27]), indicating, thus, that other factors than T may be involved in modulating the mortality impacts (see below).

The temperature conditions associated with T anomalies may be related to different biological mechanisms resulting in the death (total or partial) of organisms. In relation to long-term anomalies, the highest mortality rates were observed when long periods of warm Ts occurred at the end of the summer. Mediterranean suspension feeder species exhibit energetic constrains during summer [36], since these organisms have to cope with high respiration demands because of warm T during a period of food scarcity [16]. When these conditions are prolonged, as in the case of the years with anomalies, the organisms can suffer physiological stress that can ultimately lead to partial or total death of some specimens [16]. In previous studies, experimental data demonstrated that long duration exposure to warm T (∼23°C for >40 days), similar to the conditions observed during long-term anomalies, could cause the appearance of the firsts signs of necrosis [16]. In the same experiment, when the colonies were fed, the time of exposure to warm T before observing necrosis almost doubled, clearly indicating that feeding helps to cope with physiological stress [16]. Therefore, the physiological status of organisms is important in modulating their response to thermal stress. This factor may be behind the observed differential mortality observed during long-term anomalies in Riou and Port-Cros in 1999 and Medes (2003 and 2006).

The high T observed during short-term anomalies reached lethal levels for the benthic species of the study regions, as demonstrated through thermotolerance experiments with different Mediterranean gorgonian species ([37], [38], [39], Crisci et al., unpublished data). Additionally, the high intra-day and intra-period variability that characterized this type of anomaly could be an additional stress factor on the organisms, as found in some tropical coral species [40], [41]. Nevertheless, when this type of anomaly occurred at the beginning of summer, such as those that took place in 2006, they resulted in less severe impacts on these populations, probably because the species were less affected by energetic constraints during this period [16].

Finally, for both types of T anomalies, the induction of mortality being provoked by thermo-dependent pathogens cannot be discarded, considering that experimental and field data demonstrated that Ts≥22°C promote pathogen virulence and/or increase host susceptibility [23], [37].

Overall, the results of this study indicated that inter-regional differences in mortality rates should be mainly attributed to differences in the T conditions recorded in each region and year with a T anomaly. However, because the populations inhabiting the studied regions were subjected to different magnitudes and timing of T anomalies, we could not determine the potential role of biological factors, such as acclimatization [42], local adaptation [43], [44] or even contemporary evolution [45], previously highlighted for other marine species [46], [47], [48]. Bearing in mind that most of the species affected by the MME inhabit different thermal regimes within the NW Mediterranean basin [22], [28] and appear to be characterized by significant genetic differentiation, even at reduced spatial scales of several meters [49], [50], it seems likely that selective processes could play a role in determining their response to T anomalies. The available experimental data suggest an inter-depth differential response to the same experimental T ([38], [50]). To further explore the role of selective processes in these phenomena, regional-scale thermotolerance experiments should be conducted.

The combination of field T data on the most extreme observed Ts of each studied region with available thermotolerance data on NW Mediterranean anthozoan species allowed us to assess the risk of suffering MMEs in the different study regions (Fig. 7). In general, the T conditions observed in Medes, which exhibits an absence of extreme T (short-term anomaly) and attenuated characteristics of long-term anomalies, do not reach values causing severe damages to these organisms. Therefore, this region could be associated with a lower risk of mortality outbreaks compared with the other regions under present T conditions. In contrast, Riou and Scandola appeared to be the regions with the highest risk, as in both cases, the anomalous T conditions reached values beyond the tolerance of the species addressed in this study. The case of Riou is interesting because it is located in one of the coldest areas of the NW Mediterranean [51]. The fact that both types of anomalies could occur in this area, combined with the shallow distribution of species affected by MMEs [20], [21] leads to an unfavorable scenario for Riou populations in the future. Scandola presented the most extreme T of short and intermediate-length periods, which makes it difficult for species affected by MMEs in shallow depth ranges to survive in this region. In fact, the absence of *P. clavata* populations at the 10 m depth in this area could indirectly suggest that T could be modulating this species' depth distribution, although effects of other environmental factors cannot be discarded (light, water motion, food availability) [52], [53]. Finally, Port-Cros occupied an intermediate position in terms of the risk of presenting MMEs, mainly because of the absence of high T episodes. In accordance with these results, the number of species affected and the incidence of mortality during MMEs showed the lowest values in Medes, followed by Port-Cros and, finally, by Riou and Scandola [20], [21], [25].

In this study, we demonstrated the utility of acquiring and analyzing high resolution T series, which allowed us to determine the main T conditions responsible for the differential mortality impacts observed in the NWM basin and to assess the risk of MMEs in the studied regions. The acquisition of new high resolution T time series in different regions of the Mediterranean (e.g., /T-MEDNet, http://t-mednet.org) will allow expanding the analysis to better characterize and understand current shifts in environmental conditions at larger spatial scales. Additionally, under the present warming scenario for the Mediterranean area [54], these data will be key components in the development of MME risk maps at the scale of the NW Mediterranean. This information is urgently needed to develop sound management and conservation strategies to face the impacts of climate change on the rich marine biodiversity in the Mediterranean region [55].
